# Vomocytosis: Too Much Booze, Base, or Calcium?

**DOI:** 10.1128/mBio.02526-19

**Published:** 2019-12-24

**Authors:** Melissa Cruz-Acuña, Noah Pacifici, Jamal S. Lewis

**Affiliations:** a Department of Biomedical Engineering, University of California, Davis, Davis, California, USA; University of Texas Health Science Center at Houston

**Keywords:** vomocytosis, macrophage, pH, phagosome, nonlytic exocytosis, *Cryptococcus neoformans*

## Abstract

Macrophages are well known for their phagocytic activity and their role in innate immune responses. Macrophages eat non-self particles, via a variety of mechanisms, and typically break down internalized cargo into small macromolecules. However, some pathogenic agents have the ability to evade this endosomal degradation through a nonlytic exocytosis process termed vomocytosis. This phenomenon has been most often studied for Cryptococcus neoformans, a yeast that causes roughly 180,000 deaths per year, primarily in immunocompromised (e.g., human immunodeficiency virus [HIV]) patients. Existing dogma purports that vomocytosis involves distinctive cellular pathways and intracellular physicochemical cues in the host cell during phagosomal maturation. Moreover, it has been observed that the immunological state of the individual and macrophage phenotype affect vomocytosis outcomes. Here we compile the current knowledge on the factors (with respect to the phagocytic cell) that promote vomocytosis of C. neoformans from macrophages.

## Abstract (precis)

Macrophages are well known for their phagocytic activity and their role in innate immune responses. Macrophages eat non-self particles, via a variety of mechanisms, and typically break down internalized cargo into small macromolecules. However, some pathogenic agents have the ability to evade this endosomal degradation through a nonlytic exocytosis process termed vomocytosis.

## INTRODUCTION: WHAT GOES IN MUST COME OUT?

**FIG 1:**
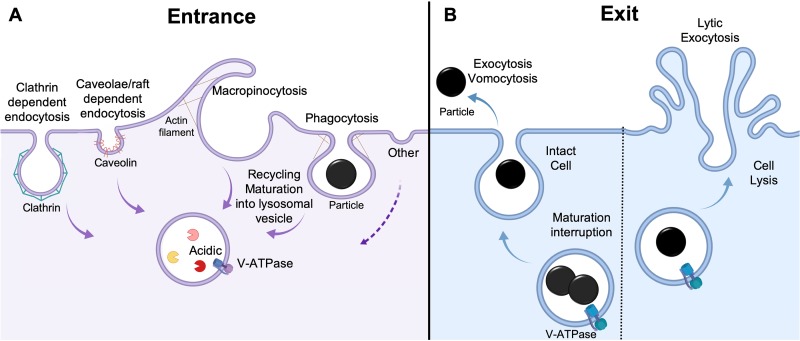
Foreign particulate entry and exit pathways into macrophages. Internalized cargo is typically directed to its degradation in a harsh, degradative environment driven by acidity and lysosomal enzyme activity. C. neoformans, however, has the ability to manipulate the progression of this process by exiting the cell via the nonlytic exocytosis mechanism, vomocytosis, which leaves the host cell intact, or by lytic exocytosis, which results in the lysis of host cell. Depicted are the types of internalization mechanisms that have been observed in macrophages. Their relative frequency of occurrence is not represented here or discussed in this article. This figure was created with BioRender.

Phagocytosis by innate immune cells is important for cell-to-cell communication, metabolism, homeostasis, and organism survival (1–4). Macrophages are critical players in the innate immune host defense system that recognize, internalize, and neutralize foreign bodies (5). These specialized cells use a variety of mechanisms, which are often concurrent and intertwined, to internalize particulate matter. Some of the best-characterized uptake pathways in macrophages include (i) clathrin-mediated endocytosis, (ii) caveolae/raft-dependent endocytosis, (iii) macropinocytosis, (iv) micropinocytosis, and (v) phagocytosis (Fig. 1A). The specific entry process depends on both the physicochemical and biological properties of the particulate (6–9). Accordingly, the intracellular fate of internalized cargo is influenced by the type of internalization executed and nature of the internalized particulate (6, 8–10).

In the case of a fungal infection, macrophages are among the early immune cell responders that typically internalize and clear fungal cells. This clearance involves the accumulation of these phagocytes at the site of infection, recognition of cell surface, fungus-specific characteristics, internalization, and degradation in an acerbic vacuole, called the phagolysosome (6, 11). However, some fungi, such as Cryptococcus neoformans, are capable of not only skirting innate immune actions but also escaping from the grasp of these phagocytic cells after capture.

C. neoformans is a globally distributed and free-living basidiomycete, mostly found residing within the decaying wood within tree trunk hollows and avian excreta (12). Therefore, this species’ survival does not depend on infection of animal hosts. However, the ease of exposure to C. neoformans, presumably by inhalation of infectious particles, resulted in an estimated 220,000 cryptococcal meningitis cases in people living with HIV/AIDS in 2017 (13). This organism has developed phagocyte escape capabilities that contribute to its virulence within immunocompromised hosts. Some theorize that C. neoformans may have developed these defenses against phagocytes in response to pressure from environmental amoebae (14). However, the amoeba Dictyostelium discoideum was shown to expel phagocytosed C. neoformans primarily via Wiskott-Aldrich syndrome protein and Scar homolog (WASH)-mediated constitutive exocytosis—an 80-min-long process involving actin restructuring and membrane recycling pathways, which is distinct from vomocytosis (15). When WASH-mediated exocytosis is blocked, a secondary, vomocytosis-like route of escape appears, characterized as a stochastic, nonlytic exocytosis process that takes place over several hours. C. neoformans can evade amoebae using constitutive exocytosis alone; therefore, the existence of vomocytosis as a secondary exit process seems evolutionarily redundant. The true evolutionary driver for this phenomenon has yet to be discovered. Vomocytosis (often referred to as nonlytic exocytosis) has also been observed in C. neoformans*-*infected macrophages. After expulsion from innate immune cells, C. neoformans is carried in the bloodstream and disseminated to the brain (16).

In this review, we highlight the biological, chemical, and physical changes within the phagocytic cell that are connected to vomocytosis. As a postscript, we discuss the potential contributions of engineering to the study of this incredible behavior and the prospective exploitation of this phenomenon for advances in biotechnology and medicine.

## ESCAPE FROM ALCATRAZ: BREAKOUT FROM MACROPHAGES

Most mammalian cells not only are capable of internalizing materials but also have built-in mechanisms to deliver intracellular contents externally. Vacuolar contents can exit the cell via exocytosis, a mechanism in which a vacuole fuses with the cell membrane, releasing its contents outside the cell (17). Exocytosis is particularly relevant in macrophages for a variety of intrinsic functions, such as phagocytosis and inflammation, and also play a role in the diseased state (18–21). For instance, exocytosis of intracellular compartments is required for phagosome formation in order to compensate for the membrane utilized in phagocytosis, a dynamin-dependent process called focal exocytosis (22). The secretion of the cytokine tumor necrosis factor (TNF) via secretory carrier membrane protein 5 (SCAMP5) is another example of exocytosis (23). Moreover, lysosomal enzymes delivered via exocytosis are also reported in the initial degradation of dying or dead adipocytes in obesity and in tumorigenic cells clearance, processes called exophagy and heterocytolysis, respectively (19, 20). Some internalized pathogens, such as C. neoformans, Candida albicans, and Cryptococcus gattii, among others, have developed a mechanism to fuse their containment vacuoles with the cell membrane, evading lysosomal degradation (24–26).

C. neoformans has developed unique lytic and nonlytic exocytosis mechanisms to escape from phagolysosome degradation (Fig. 1B). Several studies have identified virulence factors that promote escape, such as capsule shedding, laccase, and phospholipase B1 (27–31). These observations suggest that these factors may contribute to modifying host signaling events. Early studies on phagosomal escape demonstrated that C. neoformans and the phagosome can be colocalized with phagosomal maturation indicators such as major histocompatibility complex class II (MHC-II), CD63 (32, 33), and LAMP-1 (26, 33), suggesting that the phagosome is at least partially matured. However, more recent studies have identified phagosome maturation disruption at the protein and physicochemical levels (34–36). Another mechanism of escape for C. neoformans is cell-to-cell transfer or dragotcytosis. Together, these exit mechanisms, which are further discussed below, aid in C. neoformans survival and dissemination in human hosts.

### Lytic exocytosis.

The lysis of host cells is an important route of escape from the intracellular environment for many pathogens (12, 37). However, pore-forming proteins, which are commonly used by other pathogens to lyse host cells, have not been identified in C. neoformans. Studies suggest that C. neoformans mechanically disrupts host cells through proliferation within the phagosome and possibly via production of large amounts of polysaccharide capsule (38, 39). Macrophages can undergo apoptosis in response to intracellular cryptococcal signaling via the alternative NF-κB pathway (40). Further, macrophage lysis in response to intracellular C. neoformans has recently been linked to phagosome membrane permeabilization and apoptosis (36).

### Vomocytosis.

Among the different exit mechanisms, vomocytosis (or nonlytic exocytosis) by C. neoformans has been the best studied. In vomocytosis, a live fungal cell is expelled from the phagosome while keeping the host cell intact (26, 41). Similar phagocytic escape has been described for Candida albicans (25), *Chlamydia* spp. (42), Orientia tsutsugamushi (43), and Cryptococcus gattii (44, 45). However, all of these species have distinguishing features to their vomocytic mechanisms. In vomocytosis, the phagosome fuses with the plasma membrane, releasing the cryptococcal cell (46). In recent years, studies have demonstrated that this mechanism is highly regulated and driven by C. neoformans, since the heat-killed pathogen is unable to promote vomocytosis (26, 47).

### Dragotcytosis/cell-to-cell transfer.

Vomocytosis followed by phagocytosis by a nearby cell has been characterized as a new C. neoformans phagosomal escape process called dragotcytosis (48). The distinct feature of this mechanism is that there is interaction between the donor and acceptor macrophages prior to and shortly after the pathogen transfer event. Further molecular studies are needed to elucidate the cross talk between macrophages and the internalized C. neoformans during dragotcytosis.

It is noteworthy that the escape of C. neoformans from phagocytic cells via the last two described processes, vomocytosis and dragotcytosis, potentially explains how C. neoformans may exploit phagocytes to penetrate the blood-brain barrier (BBB) in a Trojan horse manner (49, 50). In previous studies, C. neoformans was detected inside phagocytes on the outer side of a meningeal capillary, which suggests that C. neoformans may have been transported within circulating phagocytes (51, 52). Moreover, Santiago-Tirado and coworkers demonstrated the occurrence of this mechanism *in vitro* (16). However, due to the difficulty of studying this mechanism *in vivo*, the extent to which C. neoformans may use the Trojan horse dissemination model to traverse into the brain remains unknown.

### Transcytosis.

Transcytosis, possibly an indirect innate immune system evasion, is the BBB cell penetration mechanism employed most by C. neoformans, which takes advantage of cellular endocytosis (53, 54). Transcytosis of the BBB has been widely demonstrated *in vitro* by showing the ability of C. neoformans to adhere to one or more receptors on the endothelial cell barrier (55, 56). The process causes marked morphological changes in the host cell, including membrane ruffling, irregular nuclear morphology, and swelling of the mitochondria and the endoplasmic reticulum (ER) (57). Studies suggest that transcytosis involves the migration of C. neoformans across the BBB in a glycoprotein cluster-of-differentiation (CD44)‐dependent manner. C. neoformans activates the ephrin A2 (EphA2) pathway via CD44, creating a permeable barrier that promotes the migration of C. neoformans across the BBB (58). More molecular events detailing the mechanisms underlying C. neoformans transcytosis remain to be fully resolved.

Collectively, these mechanisms of escape aid in the survival and dissemination of C. neoformans in the human body. However, the relationships between these processes are currently unknown. Converse to phagocytosis, many questions remain over vomocytosis, which was only identified in 2006 (26). Below, we discuss the characterized features of macrophages that have been linked to vomocytosis of C. neoformans.

### Vomocytosis involves cytoskeletal remodeling.

The cytoskeleton has a prominent role in phagocytosis, and therefore, its relevance in C. neoformans expulsion should not be surprising. Reports have shown in detail how changes in the cytoskeleton promote pathogen survival and innate immune evasion (26, 46, 59). For instance, Johnston and May found via three-dimensional confocal time-lapse imaging *in vitro* (46) that there is a dynamic and repeated actin coat assembly around C. neoformans-containing phagosomes. They also observed that vomocytosis occurred via the fusion of phagosomal compartments and plasma membrane in J774 cells (a macrophage-like cell line). This event was preceded by phagosomal permeabilization and followed by actin coat assembly. When modulating actin polymerization they found that “flash” (actin polymerization cycle) events are inversely related to nonlytic exocytosis, suggesting its role in temporarily inhibiting vomocytosis.

## PROTEIN NETWORKS INVOLVED IN VOMOCYTOSIS

### Rab GTPase protein family.

**FIG 2:**
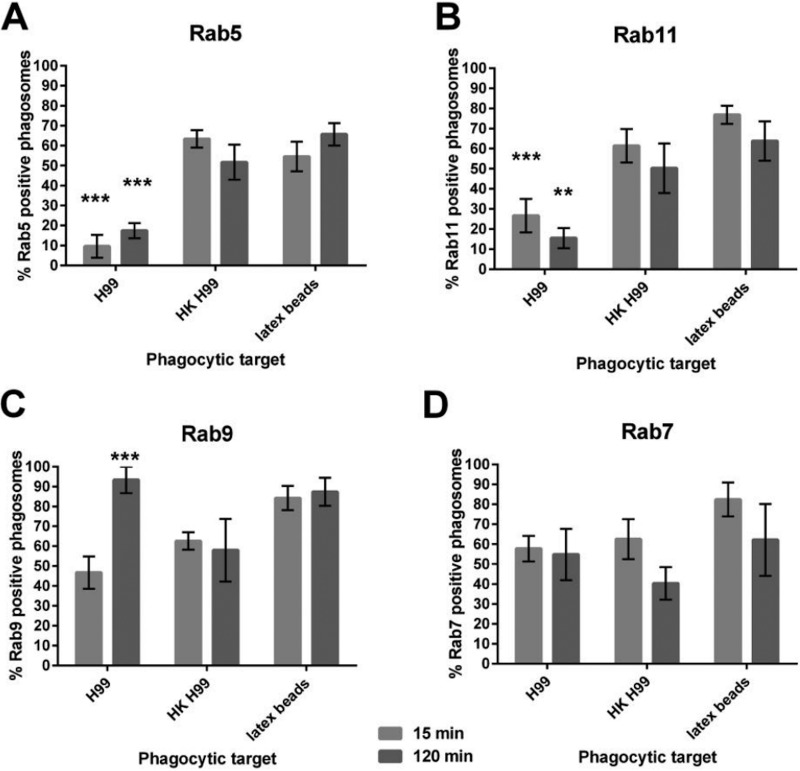
Recruitment of Rab GTPases onto the *Cryptococcus*-containing phagosome. Immunofluorescence analysis was conducted at 15 min and 2 h to detect Rab5 (A), Rab11 (B), Rab9 (C), and Rab7 (D) recruitment to phagosomes containing live C. neoformans on J774 cells. This figure has been adapted from reference 34, and the data were described therein.

Members of the Rab GTPase protein family are integral players in phagosomal maturation (60, 61). Rab5 is an early phagosome marker that recruits for the progression of phagosomal maturation. Another early phagosome marker is Rab11, which is relevant for phagosome fusion and fission with other intracellular compartments by contributing to the dynein-dependent transport of recycling endosomes. Rab9 and Rab7 are proteins associated with the late phagosomal stage. The former is a marker of endoplasmic reticulum-derived membrane, and the latter plays an essential role in the fusion of the late phagosome with a lysosome to form the phagolysosome (61). Smith et al. demonstrated that live C. neoformans promotes the rapid removal of the early phagosome markers Rab5 and Rab11 in J774 cells (Fig. 2) (34). This removal did not occur when infecting macrophages with the heat-killed pathogen or inert beads. Additionally, Rab9 levels were significantly higher on phagosomes containing live C. neoformans 2 h after phagocytosis than on phagosomes containing heat-killed pathogen and latex beads. This report demonstrated how C. neoformans affects phagosomal maturation at the protein level.

### ERK5 signaling modulation.

Another host protein that has been identified to play a role in vomocytosis, as well as in phagocytosis, is extracellular-signal-regulated kinase 5 (ERK5). At the cellular level, the ERK5 pathway is required for colony-stimulating factor 1 (CSF-1)-induced proliferation and is linked to cell metabolism in macrophages (62–65). Gilbert et al. demonstrated its critical role in suppressing the frequency of vomocytic events (64). Pharmacological inhibition and genetic manipulation of ERK5 activity both significantly raise vomocytosis rates in human macrophages, whereas stimulation of the ERK5 signaling pathway inhibits vomocytosis. Using a zebrafish model of cryptococcal disease, this study showed that reducing ERK5 activity *in vivo* stimulates vomocytosis and results in reduced dissemination of infection, likely due to expulsion before macrophage migration (limiting the chance for Trojan horse transport). Interestingly, modulation of the ERK5 signaling pathway did not induce expulsion of heat-killed C. neoformans or inert beads, suggesting the active role of the pathogen and a more complex molecular mechanism. Notably, ERK5 inhibition suppressed M2 macrophage polarization in J774 cells and human monocyte-derived macrophages (HMDMs) (64), suggesting an interesting dynamic between macrophage polarization and vomocytosis.

### Cathepsin activity.

Cathepsins are hydrolytic enzymes that have optimal activity under acidic conditions. They are important players in phagosomal maturation and are also implicated in inflammasome activation. The NLRP3 inflammasome is a multimeric protein complex important for infection eradication via the activation of interleukin 1β (IL-1β) and IL-18, which has a central role in host defense (66). In a study by Lei et al., C. neoformans biofilm was found to stimulate NLRP3 inflammasome activation in human monocytic THP-1 cells. Moreover, an increased rate of death was observed in C. neoformans biofilm-infected mice if there was no activation of this protein complex (67). Further, this group demonstrated that inhibition of cathepsin B resulted in IL-1β activation interruption in a dose-dependent manner on cells. While this report showed the importance of cathepsin B in the activation of host protection against C. neoformans *in vivo*, Smith et al. showed that macrophage infection with C. neoformans results in lack of cathepsin L activation, which is a phagosome maturation late-stage marker. In the latter study, heat-killed C. neoformans had significant activation of cathepsin L (34). There is no clear link between vomocytosis and cathepsins. However, these studies demonstrate that this class of proteins may play a role in this special event, as cathepsin activity is affected by C. neoformans infection. Moreover, these studies imply a role for phagosomal pH and other physicochemical factors which influence cathepsin activation in vomocytosis.

### Annexin A2 expression.

Annexin A2 is a membrane-bound protein involved in many processes, including phagocytosis, endocytosis, and exocytosis. Gene expression for this protein is upregulated in brain endothelial cells during transmigration of C. neoformans (55). Therefore, Stukes et al. investigated the role of annexin A2 in the interaction between macrophages and C. neoformans (68). Murine bone marrow-derived cells (BMDMs) were harvested and grown from wild-type mice and annexin A2 knockout mice. The investigators found that annexin A2-deficient macrophages exhibited lower rates of phagocytosis, a lower frequency of vomocytosis, and higher occurrences of lytic exocytosis than did wild-type macrophages. Other notable observations of infected annexin A2-deficient macrophages included an increase in C. neoformans capsule size, lower production of reactive oxygen species, and decreased levels of LC3 in phagosomes. These results align with previous findings that free radicals can damage and reduce the C. neoformans capsule (69). Stukes and coworkers also tested the significance of annexin A2 during infection, by infecting wild-type and annexin A2 knockout mice with C. neoformans. The A2 knockout mice had lower rates of survival, suggesting that this protein is important in controlling fungal infections, with vomocytosis potentially protecting macrophages from eventual lysis. These findings highlighted the role of annexin A2 in phagocytosis, antifungal defense mechanisms, and vomocytosis. While the exact underlying mechanisms of this protein’s involvement are unclear, the authors theorized that during vomocytosis annexin A2 complexes with its known binding partner, fusogenic protein SNAP-23, promote fusion between the phagosome and plasma membrane (70). Furthermore, the Ca^2+^ dependence of annexin A2 could be linked to the intracellular calcium changes observed during vomocytosis.

## PHYSICOCHEMICAL CHARACTERISTICS OF VOMOCYTOSIS

### Phagosomal pH.

An important characteristic of phagosomal maturation is its acidification. Several studies have demonstrated the ability of C. neoformans to affect the pH of phagosomes as an indication of phagosomal maturation inhibition. The pH in the phagosomal environment is highly regulated and controlled by vacuolar membrane ATPase activity. This protein is responsible for the acidification of the phagosomal compartment to a pH as low as 4.5. This H^+^ release is balanced by Cl^−^ anions (71, 72). Smith and coworkers demonstrated that significant acidification of the phagosome, which is distinctive of the phagolysosomal stage, occurred only on phagosomes containing heat-killed or UV-killed C. neoformans (34). Acidification was hindered by the presence of live C. neoformans in phagosomes of J774 cells and HMDMs. Moreover, inhibition of the microbicidal environment resulted in intraphagosomal cryptococcal budding and vomocytic activity of the internalized C. neoformans. These observations validated an earlier study by Nicola et al. that noted a relationship between the increase in phagosomal pH and the enhancement of vomocytosis events (73). Succinctly, they reported an increase in nonlytic exocytosis events when supplementing C. neoformans-containing macrophages with the weak bases chloroquine and ammonium chloride.

**FIG 3:**
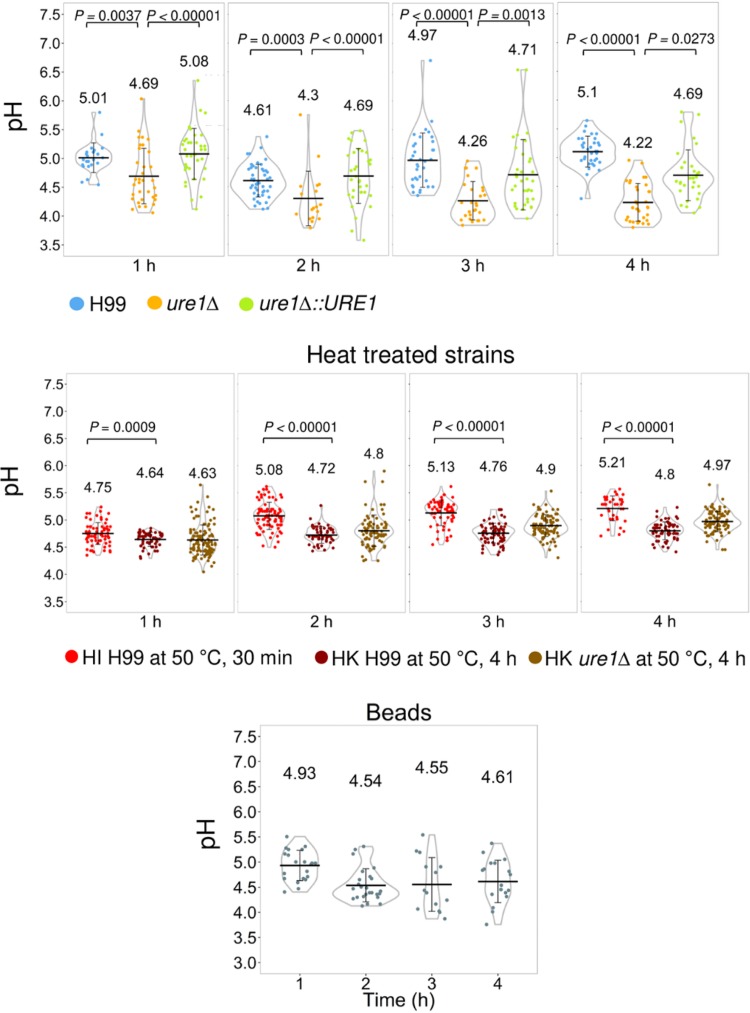
The presence of urease increases the phagolysosomal pH. BMDMs were infected with Oregon green-labeled H99, *ure1*Δ, and *ure1*Δ::*URE1* strains (top), heat-inactivated (HI) H99 (heat inactivated at 50°C for 30 min), heat-killed (HK) H99 and *ure1*Δ strains (heat killed at 50°C for 4 h) (middle), and IgG-coated polystyrene beads (bottom), and phagolysosomal pH was measured by using dual-excitation ratio fluorescence imaging at the indicated time points. Each dot represents the pH of individual phagolysosomes. The violin plot displays the probability density of data set with means (middle bar) and standard deviation. This figure has been adapted from reference 74, and the data were described therein.

Later, Fu et al. showed that production of urease was connected with increased phagolysosomal pH (74). Urease is a major virulence factor of C. neoformans upon interaction with macrophages. This enzyme breaks down urea into ammonia, which, in turn, increases the phagosomal pH in infected macrophages (75, 76). In the study by Fu and coworkers, the alkalinization of phagosomes containing C. neoformans was clearly demonstrated, and this increase in pH resulted in (i) reduced proliferation of urease-positive C. neoformans compared to a urease-negative strain, (ii) a decrease in phagolysosomal damage, and (iii) an increase in vomocytic events. An interesting finding of this study was that even coincubating J774 cells with heat-killed C. neoformans resulted in an increase in phagosomal pH in comparison to latex beads containing phagosomes (Fig. 3). The discrepancy between the latter report and what was observed in the study by Smith et al. could be due to the use of different techniques to measure pH. Fu et al. attributed the alkalinization of phagosomes containing heat-killed C. neoformans to heterogenicity in maturation of evaluated phagosomes and secretion of molecules that could affect the concentration of hydronium ions by heat-killed pathogen. It is noteworthy that they also observed that the addition of urea promoted an increase in nonlytic exocytosis events in macrophages infected with C. neoformans. Interestingly, urea also promoted nonlytic exocytosis events in urease-negative C. neoformans*-*infected macrophages. Taken together, these observations emphasize that increased phagosomal pH facilitates but does not cause vomocytosis. Moreover, vomocytosis is affected by the presence of urea and urease. Additionally, this report noted an increase in intracellular replication of C. neoformans in acidic vesicles, corroborating previous findings of C. neoformans growth under acidic conditions (77). In an *in vivo* infection model, urease-deficient C. neoformans was less virulent than wild-type C. neoformans, which reiterates the importance of vomocytosis to C. neoformans dissemination and infection.

More recently, De Leon-Rodriguez and coworkers studied the role of the C. neoformans capsule in the pH microenvironment of the phagosome (35). They hypothesized that glucuronic acid residues in the capsular polysaccharide had buffering capacity in the phagosome. They infected BMDMs with nonencapsulated C. neoformans organisms that had been previously coated with different amounts of encapsulated C. neoformans conditioned medium, which resulted in the attachment of soluble polysaccharide to their surface. More coating on nonencapsulated cells promoted an increase in phagosomal pH compared to phagosomes infected with encapsulated C. neoformans. With these findings, they suggested that the presence of glucuronic acid residues in the capsule of C. neoformans makes the polysaccharide a weak acid capable of modulating pH in the phagosome. The capsule’s acid-base properties promote fungal cell survival in the phagosome by the buffering capacity during microbicidal conditions. While interesting, the characterization of encapsulated C. neoformans conditioned medium is necessary to elucidate if there are other factors that would be capable of affecting phagosomal pH.

### Phagosome permeabilization.

Phagosome permeabilization has been observed in several studies with C. neoformans-infected macrophages (36, 51, 78, 79). Davis and coworkers studied lysosomal permeabilization in bone marrow-derived macrophages infected by C. neoformans via flow cytometry (51). They detected significant phagosome permeabilization up to 72 h after C. neoformans infection. Conversely, no significant permeabilization was observed in macrophages infected with heat-killed C. neoformans. Since the majority of C. neoformans exocytosis occurs between 5 and 14 h after macrophage uptake (26, 41), they proposed that if permeabilization is needed for C. neoformans to escape from lysosomal degradation, C. neoformans exocytosis and C. neoformans-mediated lysosomal damage are chronologically independent mechanisms (51).

Three years later, a study by De Leon-Rodriguez et al. sought to further unravel the relationship between phagosomal pH, phagosomal membrane permeabilization (PMP), lytic exocytosis, and vomocytosis (36). They found that most C. neoformans-infected J774.16 cells experiencing PMP were positive for apoptotic markers, demonstrating a relationship between PMP and apoptosis. However, they still observed populations of live cells experiencing PMP on BMDMs. Nevertheless, their observations demonstrated that macrophages undergoing apoptosis did not maintain an acidic phagolysosomal pH. They investigated the role of phospholipase B1, a virulence factor for both C. neoformans and C. gattii, in the C. neoformans induction of PMP. Macrophages infected with a C. neoformans Δ*plb1* mutant had a decrease in PMP compared to those infected with wild-type and phospholipase B1-complemented strains, suggesting a mechanism of action for this virulence factor. However, when evaluating if phospholipase B1 deficiency affected phagosomal pH, their data suggested that pH was unchanged. Induction of PMP with ciprofloxacin, a membrane-permeabilizing agent, enhanced macrophages to trigger lytic exocytosis in apoptotic BMDMs. On the other hand, vomocytic events were common in cell populations without PMP (36). Vomocytosis occurs with a frequency of 10 to 30% in macrophages and can be modulated by increases in phagosomal pH with ammonium chloride and chloroquine (73). Interestingly, these two compounds not only raise the phagolysosomal pH but also build osmotic pressure across the phagolysosomal membrane through the proton sponge effect, thereby affecting its permeability (80, 81). De Leon-Rodriguez and coworkers showed that chemical induction of PMP in C. neoformans-infected macrophages leads to a decrease of vomocytic events to mainly lytic exocytosis, signifying that vomocytosis occurs when there is no PMP. The basis of this inference is that the frequency of nonlytic exocytosis peaks before 4 h, when most macrophages still have intact phagolysosomal membranes. However, they failed to account for dragotcytosis (82).

### Calcium transport.

**FIG 4:**
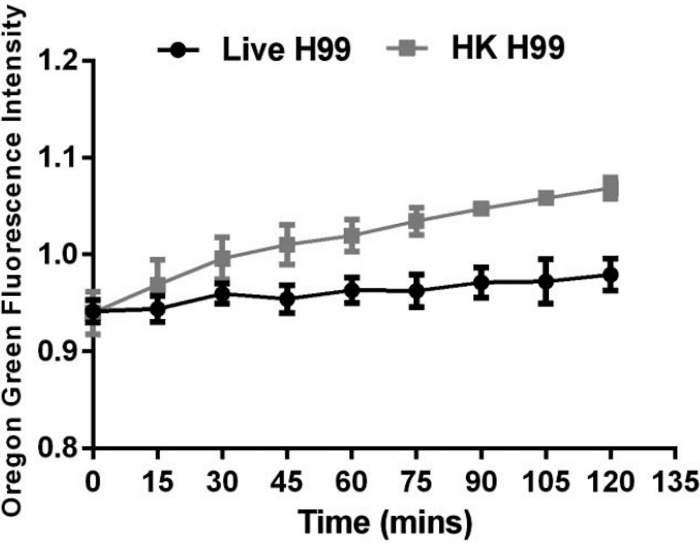
Phagosomes containing live C. neoformans do not accumulate calcium, in contrast to phagosomes containing heat-killed C. neoformans. Fluorescence intensity data from each cryptococcus-containing phagosome (live C. neoformans H99 or heat-killed H99 [HK H99]) were normalized to a randomly selected region of cytoplasm within the same cell preloaded with Oregon green BAPTA-1 1 h before infection. Data displayed are fluorescence intensity relative to that of cytoplasm, sampled every 15 min over the course of a time-lapse experiment; values are means ± SEM (*n* < 35 phagosomes) across three biological repeats. Data are as described previously (34).

Calcium ions (Ca^2+^) are critical second messengers that regulate key signaling pathways in eukaryotic cells. In the context of phagocytosis, Ca^2+^ elevations are necessary for efficient ingestion of foreign particles by some phagocytic receptors and subsequent phagosomal maturation. Ca^2+^ is required for the solubilization of the actin meshwork that surrounds nascent phagosomes, for the fusion of phagosomes with granules containing lytic enzymes, for the assembly and activation of the superoxide-generating NADPH oxidase complex, and for exocytosis (83–89). Given the role of Ca^2+^ ions in these related phenomena, some studies have delved into its role in vomocytosis. For instance, Smith et al. investigated the effect of C. neoformans on phagosomal and cytosolic calcium levels after infection (34). They observed reduced levels of Ca^2+^ in phagosomes containing C. neoformans compared to cytosolic levels up to 120 min after infection (Fig. 4). Interestingly, they found that phagosomes that contained heat-killed C. neoformans had greater calcium concentrations than cytosolic Ca^2+^ levels after infection (34). Although they did not study the relationship between phagosomal Ca^2+^ levels and vomocytic events, this is a good indication of the relevance calcium may have in this process.

In a later study, Samantaray et al. investigated the relationship between phagosomal calcium levels and C. neoformans survival and proliferation via the use of the L-type calcium channel blocker fendiline hydrochloride (90). This drug is thought to trigger endoplasmic reticulum calcium store release, thus elevating cytosolic Ca^2+^ and its signaling pathways (91, 92). They found that C. neoformans*-*containing phagosomes acidify when macrophages are exposed to the drug. This acidification promoted C. neoformans death and decreased the proliferation rate. Thus, enhancement of calcium intracellular signaling pathways promoted phagosomal maturation. The investigators did not specifically determine phagosomal pH of experimental groups, which makes it difficult to relate these results with previous discussed studies that demonstrate an increase in C. neoformans proliferation under acidic phagosomal conditions. Nevertheless, the studies by Samantaray et al. and Smith et al. make evident that Ca^2+^ influx-dependent signaling is affected by C. neoformans and that the pathogen’s virulence potential can be modulated by manipulating Ca^2+^ concentrations.

## EFFECT OF IMMUNE STATE ON VOMOCYTOSIS

### Viral infection and type I interferon.

Cryptococcus neoformans primarily infects immunocompromised hosts, such as HIV patients. Seoane et al. investigated the effect of viral infection on vomocytosis (93). They found that infection with HIV or measles virus significantly boosts the frequency of vomocytosis in C. neoformans-containing human monocyte-derived macrophages while leaving phagocytic uptake and intracellular fungal proliferation rates unaffected. Poly(I·C), a potent Toll-like receptor 3 (TLR3) agonist that induces antiviral responses in macrophages, alone was also shown to increase vomocytosis rates. These findings suggest that the macrophage response to viral infection, rather than active viral pathogenicity, is the factor that modulates vomocytosis. Furthermore, since viral infections are known for inducing expression of type I interferons (IFNs), such as IFN-α and IFN-β, these researchers tested how stimulation with IFN-α affects vomocytosis rates. Addition of IFN-α to non-virally infected macrophages was shown to produce an enhancement of vomocytosis frequency comparable to that of the virally infected group. Moreover, pharmacological inhibition of type I interferon receptors blocked the increase of vomocytosis during viral infections, confirming the role of type I interferon signaling in modulating this process. This study shows that vomocytosis likely occurs at a high rate in HIV patients and is likely linked to pathways involving type I interferon receptors.

### Cytokines.

Many cytokines have been investigated for their effect on the interaction between macrophages and C. neoformans. Various studies on Th1 cytokines IFN-γ (94), IL-12 (95), and TNF-α (96) demonstrated significantly increased fungal control in mouse models of C. neoformans infection. Additionally, Th17 cytokines IL-17 (97) and IL-23 (98) are linked to fungal protection in C. neoformans-infected mice. Conversely, Th2 cytokines IL-4 (99) and IL-13 (100) promote fungal disease progression in mice. However, none of the authors of these studies linked their observations to vomocytosis.

Therefore, Voelz et al. tested the different T helper cell cytokines for their effect on infected murine J774 cells and primary monocyte-derived human macrophages (49). Macrophages were stimulated to a Th1 (IFN-γ and TNF-α), Th2 (IL-4 and IL-13), or Th17 (IL-17) state. Interestingly, Th2 cytokines caused higher rates of intracellular C. neoformans proliferation and lower expulsion rates. Th1 and Th17 cytokines, on the other hand, reduced intracellular proliferation and caused comparably higher expulsion rates. Voelz et al. theorize that these distinct T helper cytokines modify the phagosome to result in different C. neoformans fates—intracellular proliferation (Th2) or expulsion (Th1 and Th17). The Th2 cytokines IL-4 and IL-13 have been linked to increased iron availability in macrophages (101); these metal ions could play a role in the characteristics of C. neoformans in the phagosome.

The last observation of vomocytosis rate in macrophages treated with Th2 cytokines for M2 polarization is in agreement with the aforementioned study by Gilbert et al. (64), in which higher vomocytosis rates were observed in ERK5-inhibited macrophages, along with reduction of their M2 polarization markers. ERK5 inhibition in macrophages led to a modified inflammatory profile that prevented anti-inflammatory polarization without modifying their response to inflammatory stimuli. They also observed a decrease in the presence of actin filament ruffles on macrophages treated with ERK5 inhibitor. Concisely, Gilbert et al. suggested that vomocytosis enhancement by ERK5 inhibition was a result of reduced M2 macrophage polarization and decline in actin filament ruffle formation (64).

## CONCLUSIONS AND FUTURE PERSPECTIVES

Vomocytosis is a fascinating mechanism that allows select pathogens to escape from phagocyte degradation while keeping the host cell alive. Specifically, the fungal pathogen C. neoformans has developed this mechanism, which boosts its survival in the human body. As of today, researchers have identified distinct biological, immunological, and physicochemical factors involved in this phagolysosomal escape event.

**FIG 5:**
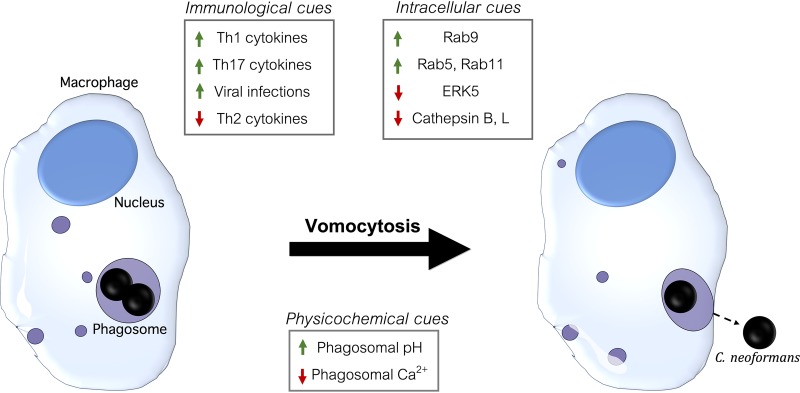
Vomocytosis occurrence is characterized by distinct intracellular, physicochemical, and immunological factors. Rab GTPase recruitment to phagosomes, which affects phagosomal maturation and cathepsin activity, is relevant for the occurrence of vomocytosis. Moreover, the manipulation of the ERK5 signaling pathway, annexin A2, phagosomal pH, and Ca^2+^ fluxes is capable of promoting vomocytosis occurrence. Finally, the immunological state of the individual and macrophage phenotype can affect vomocytosis. The arrows point out if the presence of each factor promotes or inhibits vomocytic events.

In terms of biological factors, the protein composition of the phagosome is highly altered prior to and during the expulsion of C. neoformans (Fig. 5). Early phagosomes containing live C. neoformans show a lack of Rab5 and Rab11 endosomal markers and significantly more Rab9 marker at late phagosomal stages than their counterparts containing heat-killed cells or latex beads. Also, a lack of cathepsin activity is observed in C. neoformans-containing phagosomes. These findings implicate the strong inhibition of phagosome maturation in macrophages that are actively vomocytosing cells. Other host proteins involved in vomocytosis include the mitogen-activated protein (MAP) kinase ERK5 and the membrane phospholipid binding protein annexin A2, but their exact role in this process is unknown. Outside of biological factors within the cells, the immune state of this host has shown a strong effect on vomocytosis. This is unsurprising since vomocytosis has been observed in macrophages and the phagosome is a crucial component of the innate immune system. Specifically, viral infections, Th1/2/17 cytokine exposure, and macrophage M1/M2 polarization all affect vomocytic rates. Lastly, vomocytosis has been linked to alterations in the physicochemical factors in the phagosome. For instance, Ca^2+^ levels in C. neoformans-containing phagosomes decline, relative to the cytosol, during vomocytosis. Also, the pH in the phagosome (due to urease activity and capsule acid-base properties) or in the external media influences the frequency of vomocytosis. These physicochemical factors may also contribute to rates of PMP, which are linked to decreased vomocytosis rates and increased probability for lytic exocytosis.

While we have advanced our understanding of this phenomenon, revisiting its science using fresh technologies and an interdisciplinary approach could prove knowledge rich. For instance, the relationship between macrophage polarization and vomocytosis was explored 10 years ago (49). However, current technological advances could allow us to understand, in more depth, the complexity of macrophage phenotypes and their role in disease prevention and progression. Newer, next-generation sequencing technologies could provide new insights on the transcriptional and epigenetic profile of the infected host and fungal cells at different times after infection. This information could facilitate the identification of key signaling pathways in infection and vomocytosis (as well as lytic exocytosis). Moreover, the gene expression profile of pathogens during this phenomenon could inspire novel strategies to manipulate phagosomal escape for a variety of purposes, including the design of drugs to combat the infection or mimic vomocytosis (102). Some physicochemical changes that occur during vomocytosis (intracellular Ca^2+^ concentration, direct phagosomal pH modulation, and PMP) have been characterized. However, there are still challenges in understanding their relationships to vomocytosis. Moreover, there are other key phagosomal physicochemical features that have yet to be investigated with respect to vomocytosis (e.g., oxygen ions). New technology to accurately record and report this event could also be instrumental for new findings on vomocytosis. Most studies on vomocytosis use time-lapse microscopy as a quantitative and monitoring tool, which is quite labor-intensive and inaccurate. Engineering new reporter systems to study vomocytosis will help to elucidate the interplay between biological, physical, and chemical factors that influence this behavior. Similarly, accurate measurement of vomocytosis-influencing factors can draw links to the intracellular fate of phagocytosed C. neoformans in macrophages. This knowledge will potentially help in designing treatments that target this fungal pathogen. Excitingly, a comprehensive view of vomocytosis can also lead to the development of biomimetic drugs that evade the innate immune system for improved therapeutic outcomes in a plethora of diseases.
